# Problems and prospects of current studies on the microecology of tongue coating

**DOI:** 10.1186/1749-8546-9-9

**Published:** 2014-03-05

**Authors:** Juan Ye, Xueting Cai, Peng Cao

**Affiliations:** 1 Jiangsu Branch of China Academy of Chinese Medical Sciences, Nanjing 210028, China; 2 Laboratory of Cellular and Molecular Biology, Jiangsu Province Institute of Traditional Chinese Medicine, Jiangsu, China

## Abstract

Tongue diagnosis in traditional Chinese medicine (TCM) assesses the health by investigation of tongue coating. The science and technology of tongue coating analysis have become a significant issue for modernization of TCM. The relationship between microecology of tongue coating and TCM was relevant to the syndrome differentiation in TCM, such as the *cold*/*hot* syndrome may exhibit different specific microbiota patterns in the tongue coating. This article provides a review on the microbiota research of tongue coating.

## Introduction

**Figure 1:**
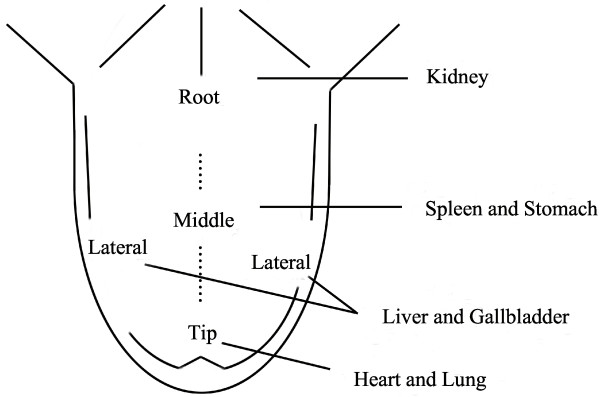
Organ layout of tongue regions.

Tongue diagnosis, as a major means of observation and a characteristic feature of traditional Chinese medicine (TCM), discriminates physiological functions and pathological conditions by observing the changes in the tongue coating. In TCM theory, the tongue is an outer extension of *pi* (*spleen*) and *wei* (*stomach*), while the tongue coating is produced by *wei qi* through fumigation [1]. The tongue coating reflected the status of *pi* and *wei*, corresponding to physiological and clinicopathological changes of inner parts of the body (Figure 1). It is the first information for TCM doctors to make a diagnosis and cannot be neglected. The status of *zang* and *fu* (internal organs), *qi*-*xue* (*blood*), and *fluids* of the human body, and the nature and severity of the disease, may be reflected in the tongue coating [2].

In Western medicine, the concepts of “geographic tongue” and “tongue colored regions” has been related to illness [3], indicating some advantages of tongue diagnosis (*e.g.*, non-invasive and simple) [4]. Inspecting plays an important role in tongue diagnosis but usually deemed as the empirical judgments by TCM doctors using their naked eyes. A tongue diagnosis is affected by examination circumstances, *e.g.*, light source, patient’s posture, and doctor’s condition, its objectivity and reproducibility has been questioned. Studies on the molecular basis of tongue diagnosis may provide significant contributions toward personalized medicine [5].

The National Institutes of Health officially launched the Human Microbiome Project in 2007, designed to determine the common core microbiome among different individuals [6]. In 2010, the European Union carried out the Human Gut Microbiome Project [7]. The microecology, the inner microecosystem, an organic integrity consisting of natural microbiota, host, and environment are interdependent and interactive of the body influences physiology and pathology [8]. The inner microecosystem contains an intercrossing network structure, among different levels and different segments, and a dynamic balance that consists of material, energy, and information flow [8]. The microecological balance is formed in dynamic physiological combination during the long-term historical evolution process [8]. However, when microecological imbalance occurs, pathological conditions arise [9]. The microbial flora on the tongue coating form one of the major microbiota in the human body, and are at the forefront of the alimentary system [10,11]. The tongue shares considerable similarity with the gut in microbial diversity [12,13]. Not only the human gut microbiome, but also the characteristics and structures of microbiomes are involved in the human health status [14,15]. Patients with different diseases might exhibit different characteristics and structures of microbiome [9,16,17].

### Recent advances

Total bacterial count on the tongue coating and the content of lysozyme were decreased after cure of acute pancreatitis (AP) [18]. The microbiota imbalance on the lingual surface was related to the changes in the tongue coating. AP patients had a thick tongue coating and increased in Gram-negative anaerobic bacilli [18]. Zhu *et al*. [19] prepared tongue coating smears for microscopic examination after Gram-staining to set the quantitative criteria for analyzing the microbiota on the tongue coating of patients with *damp-heat* syndrome. They discovered that the total bacterial count was higher in *damp-heat* syndrome’s yellow-dense tongue coating than in the normal white-greasy coating. Another study [20] diagnosed patients suffering from diarrhea-predominant irritable bowel syndrome, through microscopic examination of tongue coating smears to be a *pi* and *wei*’s *shi-re* (*damp-heat)* syndrome. Denaturing gel gradient electrophoresis (DGGE) was increasingly used in tongue coating studies. Fei *et al*. [21] discovered high similarity in DGGE analysis among different samples from the patients with lung cancer, indicating that the type of tongue coating was similar in the composition of microbial flora. 16S rRNA-DGGE was used to investigate the microbial changes in chronic gastritis patients’ greasy tongue coating, and found a new species of bacteria closely associated with the generation of greasy tongue coating [22]. The new species has a nearest neighbor *Moraxella catarrhalis* with a similarity of 96.2%. The microbial changes in the oral cavity could be one of the causes of the generation of greasy tongue coating. In addition, research on the relationship between TCM and tongue coating using the tongue coating rating scale found that a *Jianpishenshi* decoction changed the intestinal microecology and tongue coating of the patients with *pi-xue* (*spleen-deficiency*) syndrome [23].

### Research issues

First, few modern experimental techniques ‘have been employed by the studies of the microecology of tongue coating such as bacteria cultures and microscopic examination. Conventional bacteria cultures and microscopic examination of smears are inadequate to analyze large sizes of samples [24]. Second, the sample collection was not systematic [25]. The lack of objective judgment of “*zheng*” makes eliminating individual differences and differences between TCM doctors difficult. The diseases of different internal organs would be reflected in different regions (Figure 1), such as the tongue tip for *xin* (*heart*) and *fei* (*lung*) that belong to *upper-jiao*, middle tongue for *pi* and *wei* that belong to *middle-jiao*, tongue root for *shen* (*kidney*) that belongs to *lower-jiao*, and lateral tongue for *gan* (*liver*) and *dan* (*gallbladder*). It is necessary to establish a standard process for collecting tongue coating samples to ensure the comparability of samples collected from different patients.

### Prospects

**Figure 2:**
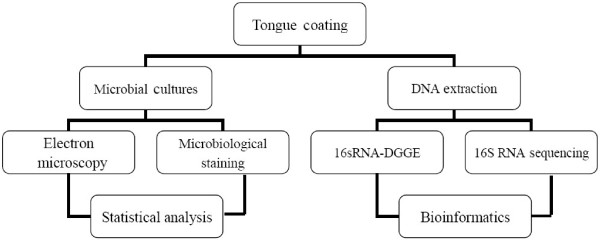
Outlook of current methodological approaches for tongue coating analyses regarding the relationship with microecology.

First, inclusion and exclusion criteria for sampling collection should be established. Second, non-culture-based bacteriological technology, like 16sRNA-DGGE [26], 2S-DGGE [27], RFLP-PCR [28], and RAPD-PCR [29], should be used for bacterial component analyses, and floral structural relation analyses, and flora network analysis (Figure 2).

Bai *et al*. [30] analyzed tongue-coating microbiomes and their relationships with tongue diagnosis, proposing a method of *zheng* discrimination before Kanawong *et al.*[31], by a machine learning algorithm with a color space set of features. Lo *et al*. [32] proposed standardization of *zheng* by determining the regions in which the samples were distributed on the tongue dorsa and fixing the sample collection region. Bai *et al*. [30] applied the next-generation sequencing methods to analyze tongue coating samples *via* bacterial 16sRNA V6 regions and analyzed the network of operational taxonomic units and microecology of *zheng*.

## Conclusions

The status of *qi*-*blood*, *cold* or *hot* syndrome, and progress of some diseases are associated with the change in microbiome on the tongue surface.

## Consent

Written informed consent was obtained from the patient’s for the publication of this report and any accompanying images.

## Abbreviations

TCM: Traditional Chinese medicine; AP: Acute pancreatitis; DGGE: Denaturing gel gradient electrophoresis.

## Competing interests

All authors declare that they have no competing interests.

## Authors’ contributions

PC conceived and designed the study. JY, CX and PC wrote the manuscript. All authors read and approved the final version of the manuscript.
